# Exploring the impact of multidecadal environmental changes on the population genetic structure of a marine primary producer

**DOI:** 10.1002/ece3.2906

**Published:** 2017-03-30

**Authors:** Nina Lundholm, Sofia Ribeiro, Anna Godhe, Lene Rostgaard Nielsen, Marianne Ellegaard

**Affiliations:** ^1^ The Natural History Museum of DenmarkUniversity of CopenhagenCopenhagen KDenmark; ^2^ Glaciology and Climate DepartmentGeological Survey of Denmark and Greenland (GEUS)Copenhagen KDenmark; ^3^ Department of Marine SciencesUniversity of GothenburgGöteborgSweden; ^4^ Deparment of Geosciences and Natural Resource ManagementUniversity of CopenhagenFrederiksbergDenmark; ^5^ Department of Plant and Environmental SciencesUniversity of CopenhagenFrederiksbergDenmark

**Keywords:** dinoflagellate, environmental change, microsatellites, phytoplankton resting stage, population genetic structure, sediment core

## Abstract

Many marine protists form resting stages that can remain viable in coastal sediments for several decades. Their long‐term survival offers the possibility to explore the impact of changes in environmental conditions on population dynamics over multidecadal time scales. Resting stages of the phototrophic dinoflagellate *Pentapharsodinium dalei* were isolated and germinated from five layers in dated sediment cores from Koljö fjord, Sweden, spanning ca. 1910–2006. This fjord has, during the last century, experienced environmental fluctuations linked to hydrographic variability mainly driven by the North Atlantic Oscillation. Population genetic analyses based on six microsatellite markers revealed high genetic diversity and suggested that samples belonged to two clusters of subpopulations that have persisted for nearly a century. We observed subpopulation shifts coinciding with changes in hydrographic conditions. The large degree of genetic diversity and the potential for both fluctuation and recovery over longer time scales documented here, may help to explain the long‐term success of aquatic protists that form resting stages.

## 1 Introduction

Marine protists are responsible for approximately half of the planetary primary production and have important roles in global biogeochemical cycles (Chavez, Messié, & Pennington, 2011; Zhao & Running, 2010); yet, our understanding of the genetic diversity, structure, and evolution of protists is still in its infancy. Marine planktonic protists are often ephemeral in the water column, have short generation times (dividing 0.3–1 times per day), and the potential to respond to short‐term changes in the environment. Many species form resting stages that ensure a larger degree of inter‐seasonal sustainability, protecting the species, and the genotypes, from extinction (e.g., Ribeiro et al., 2011). Due to their mainly asexual reproduction and potentially unlimited dispersal, protists have been considered to have large and geographically widespread populations with low genetic diversity. However, population genetic studies have revealed that aquatic protists are actually characterized by an unexpectedly high genetic diversity (e.g., Dia et al., 2014; Lowe, Montagnes, Martin, & Watts, 2010; Nagai et al., 2007; Richlen, Erdner, McCauley, Libera, & Anderson, 2012; Rynearson & Armbrust, 2000; Rynearson, Newton, & Armbrust, 2006; Tesson, Montresor, Procaccini, & Kooistra, 2014). How this diversity is maintained is one of the key questions in plankton ecology. For most species, the frequency of sexual reproduction is unknown and recent studies have shown that the effective population sizes may be relatively small compared to the enormous total population size (Watts, Lundholm, Ribeiro, & Ellegaard, 2013). Genetic differentiation related to spatial patterns has been explored in marine protists at both small (Darling, Kucera, & Wade, 2007; Godhe & Härnström, 2010; Lowe et al., 2010; Rynearson & Armbrust, 2004) and larger scales (Casteleyn et al., 2010; Iglesias‐Rodriguez, Schofield, Batley, Medlin, & Hayes, 2006).

Few studies have addressed temporal genetic differentiation in aquatic protists and most of these studies have dealt with short‐term changes, that is, within periods of days, weeks, or months (Erdner, Richlen, McCauley, & Anderson, 2011; Lebret, Kritzberg, Figueroa, & Rengefors, 2012; Richlen et al., 2012; Rynearson & Armbrust, 2005; Rynearson et al., 2006) or among consecutive years (Tesson et al., 2014). However, comparison of phenotypic and genotypic variation over decades, or even up to a century, is possible for some aquatic protists that have the capability to form resting stages that can be germinated from benthic “seed banks.” The only such long‐term study on an aquatic protist reported so far (to the best of our knowledge) showed an essentially stable genetic structure of the diatom *Skeletonema marinoi* during 100 years (Härnström, Ellegaard, Andersen, & Godhe, 2011). Determining temporal genetic diversity is important as assessments of spatial population structure and genetic diversity indirectly assume that local populations are temporally stable (Heath, Busch, Kelly, & Atagi, 2002).

**Figure 1:**
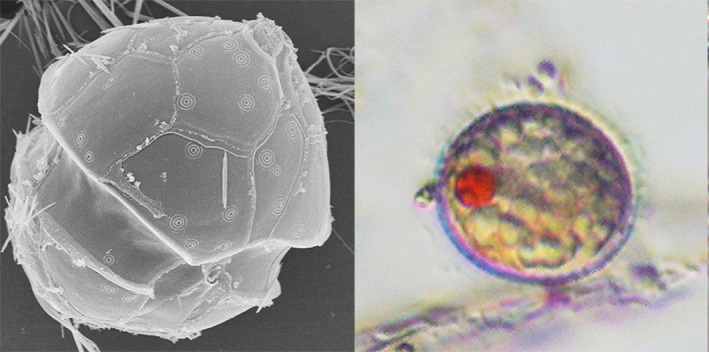
*Pentapharsodinium dalei* illustrated by an SEM micrograph of the vegetative cell, and LM micrograph of the live cyst

The marine dinoflagellate *Pentapharsodinium dalei* Indelicato and Loeblich III (Figure 1) produces resting cysts that can remain viable in coastal sediments for up to a century (Lundholm et al., 2011), offering an excellent possibility to explore long‐term stability or differentiation associated with past environmental transitions (Ribeiro, Berge, Lundholm, & Ellegaard, 2013). Today, *P. dalei* is commonly found at high latitudes (Dale, 1996; Rochon, Vernal, Turon, Matthießen, & Head, 1999), is often dominant in subpolar and polar regions, and makes up a regular component of the spring bloom in the North Atlantic and North Pacific, particularly in coastal areas (Dale, 1977; Harland, Nordberg, & Filipsson, 2004a, 2004b; Penaud et al., 2011). The cyst is often used as a palaeoecological indicator and is abundant in marine sediments in the North Atlantic region and the Arctic (e.g., Dale, 1996; Howe et al., 2010; Penaud et al., 2011; Ribeiro, Moros, Ellegaard, & Kuijpers, 2012). Thus, *P. dalei* has been found to be one of the first indicators of global warming at high latitudes, as global warming results in a longer spring period with higher production of *P. dalei* and a marked increase in *P. dalei* cysts in the sediments (Dale, 2001). In lower latitudes, it is used as indicator of colder temperatures (Dale, 1996; Rochon et al., 1999). High abundances of *P. dalei* have further been correlated with increased stratification (Harland et al., 2004a, 2004b).

We revived *P. dalei* resting cysts from dated sediment layers spanning nearly a century, established cultures of vegetative cells (Lundholm et al., 2011), amplified species‐specific microsatellite markers (Lundholm, Nielsen, Ribeiro, & Ellegaard, 2014), and explored the data for estimating protist effective populations sizes (Watts et al., 2013). The sediment cores were retrieved from Koljö Fjord, a sill fjord that has laminated, oxygen‐depleted sediment well suited for the preservation of resting stages. The fjord has experienced changes in temperature and alternating periods of low and high salinity (Filipsson & Nordberg, 2004a) that have been linked to hydrographic variability associated with the North Atlantic Oscillation (NAO; Filipsson & Nordberg, 2004b).

**Figure 2:**
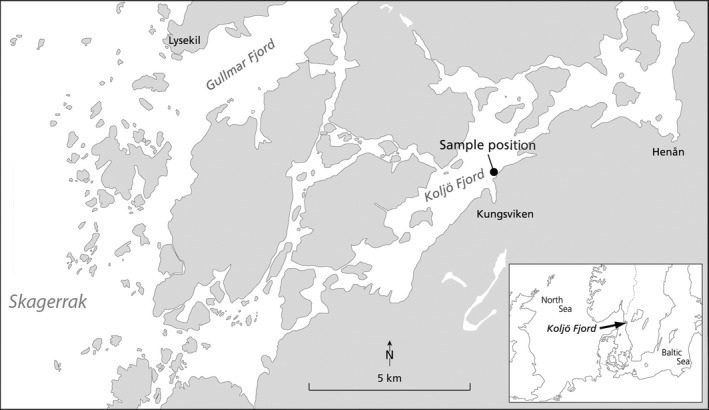
Map showing the sampling location in Koljö Fjord, Sweden

The NAO is a predominantly natural mode of atmospheric variability, which affects climate conditions in the Atlantic sector. In Koljö Fjord in Sweden (Figure 2), predominantly negative NAO conditions are associated with cold winters, increased easterly and north‐easterly winds, often the presence of sea ice cover in the fjord, high bottom water salinities, increased upwelling, a strong pycnocline, and potentially nutrient deplete surface waters(Filipsson, Björk, Harland, McQuoid, & Nordberg, 2005; Filipsson & Nordberg, 2004b; Harland et al., 2004a). Paleoecological studies in Koljö Fjord have indicated distinct changes in species dominance in the fjord around 1940 with a significant increase in dinoflagellate abundance, and a second change in dinoflagellate cyst assemblage ca 40 years later, including a marked decrease in abundance of *P. dalei* (Filipsson et al., 2005; Harland et al., 2004a). The high abundances of *P. dalei* from ca. 1940–1980 correlate with a predominantly negative mode of the winter NAO, resulting in input of nutrients at depth, and a stronger thermohaline stratification in the fjord (Nordberg, Filipsson, Gustafsson, Harland, & Roos, 2001). The sediment layers studied represented almost a century and were selected to temporally span the environmental shifts that have happened in the fjord, the effects of which we wanted to explore. Some of the sediment layers studied thus represent the period with negative NAO conditions from around 1940–1980 characterized by cold winters, a strong pycnocline with high salinities in the bottom water and potentially nutrient deplete surface waters and a relative high abundance of *P. dalei*. The remaining sediment layers were chosen to represent the positive NAO conditions before 1940 and after 1980, both characterized by mild winters and a weaker thermocline with relatively lower salinities as well as a decrease in abundance of *P. dalei* compared to 1940–1980.

The aim of this study was to explore the effects of the multidecadal environmental changes on the population genetic structure of *P. dalei*. The temporal genetic structure reveals how populations are affected by environmental factors on a multidecadal scale and may assist in projecting how primary producers will respond to future environmental and climate changes.

## 2 Materials and Methods

### 2.1 Sediment core material

Multiple sediment cores were retrieved from Koljö Fjord (58°13.793 N, 11°34.550) at 45 m water depth in April 2006 (Figure 2; Lundholm et al., 2011). This sill fjord is located on the west coast of Sweden and is part of an open‐ended fjord system connected to the Skagerrak. It has limited oxygen supply, minimum tidal activity, virtually no benthic macrofauna, and therefore generally undisturbed sediments (Nordberg et al., 2001). The cores were X‐rayed while intact and sliced in 1 cm slices used for dating and isolation of resting stages. Five sediment layers (age depths) were chosen for this study, with estimated dates of 2006, 1985 (±3 years *SE*), 1970 (±4 years *SE*), 1960 (±5 years *SE*), and 1922 (±12 years *SE*), based on 210Pb and 137Cs analyses. For details on sediment processing and dating, see Lundholm et al. (2011) and Ribeiro et al. (2011). The sediment cores from Koljö fjord were characterized by clear laminations visible in X‐radiographs, which were consistently present in all cores, indicating undisturbed sediment records and the age‐control was robust, as shown by the small associated error of each sediment layer (not illustrated; see Lundholm et al., 2011: figures 4, 6).

### 2.2 Sampling, cyst germination, and establishment of cultures

Subsamples (c. 2–5 cm^3^) of each of the five layers were placed in an ultrasonic bath for 2 min for separation of aggregated particles and wet sieved with filtered seawater through a 100‐μm sieve onto a 25‐μm metallic‐mesh calibrated sieve (Endecotts, London, UK). Separation of resting stages from the remaining sediments was performed using a solution of sodium metatungstate with a density of 1.3 g/ml and centrifugation at 1,500 *g* for 10 min (Bolch, 1997).

Single intact *P. dalei* cysts (>450 cysts in total) were isolated by micropipetting and transferred into Corning 96‐microwell plates (Corning Inc., Corning, NY, USA) each well containing 0.25 ml of TL growth medium (Larsen, Moestrup, & Pedersen, 1994). The plates were kept at 15°C, at an irradiance of 80–100 μmol photons m^−2^ s^−1^ and a light:dark cycle of 16:8 hr and were examined for germinated motile cells under an inverted microscope Olympus CKX31 and Nikon TMS on a regular basis, that is, every second to fourth day. Successful germinations were recorded and individual motile cells reisolated by micropipetting and transferred to culture flasks containing TL medium under the above conditions. We aimed at isolating 25 monoclonal strains per layer, and in total, 193 *P. dalei* strains were established (Table 1).

**Table 1:** Summary of genetic diversity across six loci for *Pentapharsodinium dalei*: sediment core depth and year, germination percentage, number of clonal strains (*M*), number of clonal strains genotyped (*N*), number of multi locus genotypes (*G*), total number of alleles (*A*), average number of alleles across loci (*N*_A_), average number of alleles across loci corrected for sample size (with rarefaction) (*N*_A correct_), richness of private alleles (with rarefaction) (NPA). Average gene diversity across loci

Sediment depth (cm)	Year	Germination percentage	*M*	*N*	*G*	*A*	*N*_A_	*N*_A correct_	NPA	Average gene diversity across loci
1	2006	28	50	21	21^a^	44	7.3	4.67	0.77	0.80 ± 0.10
10	1985 ± 3	65	42	27	27^a^	58	9.7	4.99	0.85	0.83 ± 0.06
14	1970 ± 4	45	15	8	8^a^	31	5.2	5.17	1.11	0.86 ± 0.05
20	1960 ± 5	61	39	20	20	50	8.3	5.34	1.08	0.87 ± 0.06
34	1922 ± 12	5	47	18	18^a^	36	6.0	4.35	0.42	0.80 ± 0.05

a One multi locus genotype in common.

More than one clonal culture was established from each germinated cyst. Each clonal strain originates from a single haploid cell germinated from a presumably diploid hypnozygote (Bravo & Figueroa, 2014). We used only one clonal strain from each cyst in the analyses.

### 2.3 DNA extraction and genotyping

The clonal strains were harvested by centrifugation and the material stored at −20°C. Genomic DNA was extracted using a modified CTAB‐extraction method (Lundholm, Daugbjerg, & Moestrup, 2002). To ascertain correct species identification, the ITS region (ITS1, 5.8S, ITS2) of nuclear rDNA was amplified and sequenced for randomly selected strains from each of the five layers, as described in Lundholm et al. (2014).

The samples were genotyped at six dinucleotide microsatellite loci (Pendal 3, 7, 14, 19, 23, 30) following the procedure described in Lundholm et al. (2014). The PCR products were loaded on an ABI 3730XL analyzer (Applied Biosystens) using the commercial GeneScan service (Macrogen, Korea), and allele sizes were assigned relative to the internal size standard 400HD ROX. The results were analyzed using GeneMapper 4.1 software (Applied Biosystems) where null alleles were given the code 1. The main bulk of the raw data was used for estimating effective population sizes of *P. dalei* (Watts et al., 2013), and genotypes were archived with Dryad: doi:10.5061/dryad.221t6. The dataset has been reanalyzed and some null alleles determined as belonging to one allele. This study comprises the first analyses of genetic structure for this dataset.

### 2.4 Microsatellite data analysis

As *P. dalei* is haploid, null allele frequencies were estimated by direct observation. Strains for which more than half of the loci did not amplify after at least three re‐amplifications were removed from the dataset. The absence of amplification in the remaining strains was considered to indicate the presence of null alleles. If several clonal strains isolated from one germinated cyst were successfully genotyped, one randomly chosen representative was included in the final dataset.

Genetic diversity was assessed by calculating total number of alleles, and allelic richness and private allelic richness per sample using rarefaction with the software HP‐Rare which accounts for sample size effects by calculating unbiased estimates of allelic richness (Kalinowski, 2004, 2005). Clonal diversity was calculated as the number of unique genotypes to the total number of analyzed strains. In addition, the number of genotypes and estimates of gene diversity were measured across loci for each sample. Gene diversity was based on the mean number of differences between all haplotype pairs as implemented in Arlequin 3.5.1.2 (Table 1).

Linkage disequilibrium (LD) was tested for each layer separately and globally using an exact test for LD for haplotypic data applied as implemented in Arlequin 3.5.1.2 with a Markov chain length of 10,000 and 1,000 dememorization steps for each of the age depths and Markov chain length of 1,000,000 and 100,000 dememorization steps for the global dataset. The sequential Bonferroni's procedure was used to test “table‐wide” significance levels (Holm, 1979). Pairwise genetic distance was computed using Arlequin 3.5.1.2.

### 2.5 Population structure

To assess patterns of population structure, a principal component analysis was conducted in R (version 3.0.2; R Development Core Team, 2007) using the function dudi.pca in the library Ade4 (Thioulouse & Dray, 2007). Prior to analysis, missing values were substituted by allele frequency averages. Pairwise *F*_ST_ for the five layers were calculated based on allele differences among haplotypes within and among layers using Arlequin 3.5.1.2. Significances of the estimates were tested by permuting individuals between populations 10,000 times.

Temporal genetic structure was further explored using Bayesian cluster analysis as implemented in the software STRUCTURE version 2.3.4 (Pritchard, Stephens, & Donnelly, 2000). First, the number of potential clusters (*K*) was assessed based on 20 iterations with *K* = 1–7, burn‐in length of 50,000 and additional 100,000 Markov chain Monte Carlo steps. As the five layers were temporally separated by at least 10 years, simulations were carried out with “no admixture” using sampling information a priori to assist the clustering (the LOCPRIOR model). This model is particularly informative when signals of genetic divergence are low, and when the number of loci and/or the samples size is restricted (Hubisz, Falush, Stephens, & Pritchard, 2009) and resulted in a stronger signal than without LOCPRIOR. Results were then subjected to STRUCTURE HARVESTER version 0.6.94 (Earl & von Holdt, 2012) to find the most likely *K* by estimating Δ*K* (Evanno, Regnaut, & Goudet, 2005). Final analyses were performed using the two clusters identified using STRUCTURE HARVESTER with increased burn‐in length of 100,000 and additional 1,000,000 Markov chain Monte Carlo steps and 20 iterations (both with and without LOCPRIOR) as above.

A hierarchical analysis of molecular variance (AMOVA, Excoffier, Smouse, & Quattro, 1992) was applied to test the significance of the clusters identified by STRUCTURE. AMOVA was performed in Arlequin 3.5.1.2 using Excoffier and Lischer (2010), Excoffier et al. (1992), Weir (1996), and Weir and Cockerham (1984) on the hierarchies: among individuals within samples, among samples within clusters and among clusters. The significance of the associated *F*‐statistics (*F*_SC_ and *F*_CT_) was tested using a nonparametric permutation approach (Excoffier et al., 1992) where individuals among samples within clusters, and samples among clusters, are shuffled and test statistics recalculated. A total of 16,000 permutations were carried out, and the observed test statistics was then compared to the distribution of test statistics based on permuted data.

## 3 Results

A total of 193 clonal cultures were established from more than 450 cysts of *P. dalei* individually isolated from five sediment layers down to a core depth of 34 cm (corresponding to year 1922 ± 12). Some cultures ceased to grow before a sufficient number of cells could be harvested for DNA extraction, resulting in 147 genotyped clonal strains. The layers were selected to span the known changes in NAO mode and shifts in *P. dalei* dominance from cyst records, as well as based on the number of viable cysts in different layers. The number of strains established from the lower layers represented the highest number we possibly could obtain as the entire samples were screened for viable cysts. The percentage of germinated cysts decreased downcore from a maximum of 65% (Table 1). All sequenced ITS rDNA regions of *P. dalei* strains were identical (Genbank accession number KU161681).

All primer pairs amplified a single PCR product, consistent with *P. dalei* being haploid. However, four strains yielded multiple alleles per locus indicating that a planozygote (swimming pre‐encystment cell) was genotyped or, alternatively, that two different cells from the germinating diploid cyst had been inadvertently isolated. These samples were not included in the analyses.

### 3.1 Genetic diversity and LD

All loci were polymorphic, yielding a total of 80 alleles. One locus, Pendal 14, failed to amplify in one or two clonal strains per age depth (except in 1960 and 1970) indicating the presence of null alleles. The remaining five loci amplified in all clonal strains, except one single null allele, which was observed in one of the markers (Pendal 7). The total number of alleles per locus ranged from 8 to 20 and the mean genetic diversity per locus ranged from 0.74 to 0.90. The total number of alleles observed in each sample ranged from 31 to 58 (Table 1). When corrected for sample size, the number of alleles ranged from 4.35 in year 1922 to 5.34 in year 1960. The number of alleles was higher in the clonal strains originating from 1960 to 1985 (4.99–5.34), and lower in the oldest (4.35) and the most recent (4.67) sediment layers. The private allelic richness was lowest (0.42) in the strains originating from 1922 and the most recent strains (0.77), and higher (0.85–1.11) in the group of strains established from 1960 to 1985 (Table 1). An average gene diversity of 0.80–0.87 was found across loci (0.80 for 1922 and recent samples, 0.83–0.87 for 1960–1985 samples), and *G*/*N* = 1 for all layers (Table 1).

Two multi locus genotypes were observed twice (one in layers 1922 and 1970, another in layers 1985 and 2006). Hence, 97.9% of the genotypes were unique. There was no significant difference among age depths in the frequency of nonamplifying genotypes (0.000–0.043). Tests of LD displayed no significant linkage between any loci after Bonferroni's correction.

### 3.2 Genetic differentiation and structuring

The principal component analysis of allele frequencies revealed no difference among the samples from the five layers (Fig. S1). The first two axes accounted for 17.07% of the variation in the dataset. The pairwise *F*_ST_ showed weak or no genetic differentiation among samples (Table 2) with no significant differences after Bonferroni's correction.

**Table 2:** Pairwise genetic differentiation among age depths using *F*_ST_

	2006	1985	1970	1960
1985	0.0126			
1970	0.0405	−0.0054		
1960	0.0090	−0.0116	−0.0101	
1922	−0.0161	−0.0005	0.0103	0.0046

No pairwise *F*_ST_ was significant after Bonferroni's correction.

**Figure 3:**
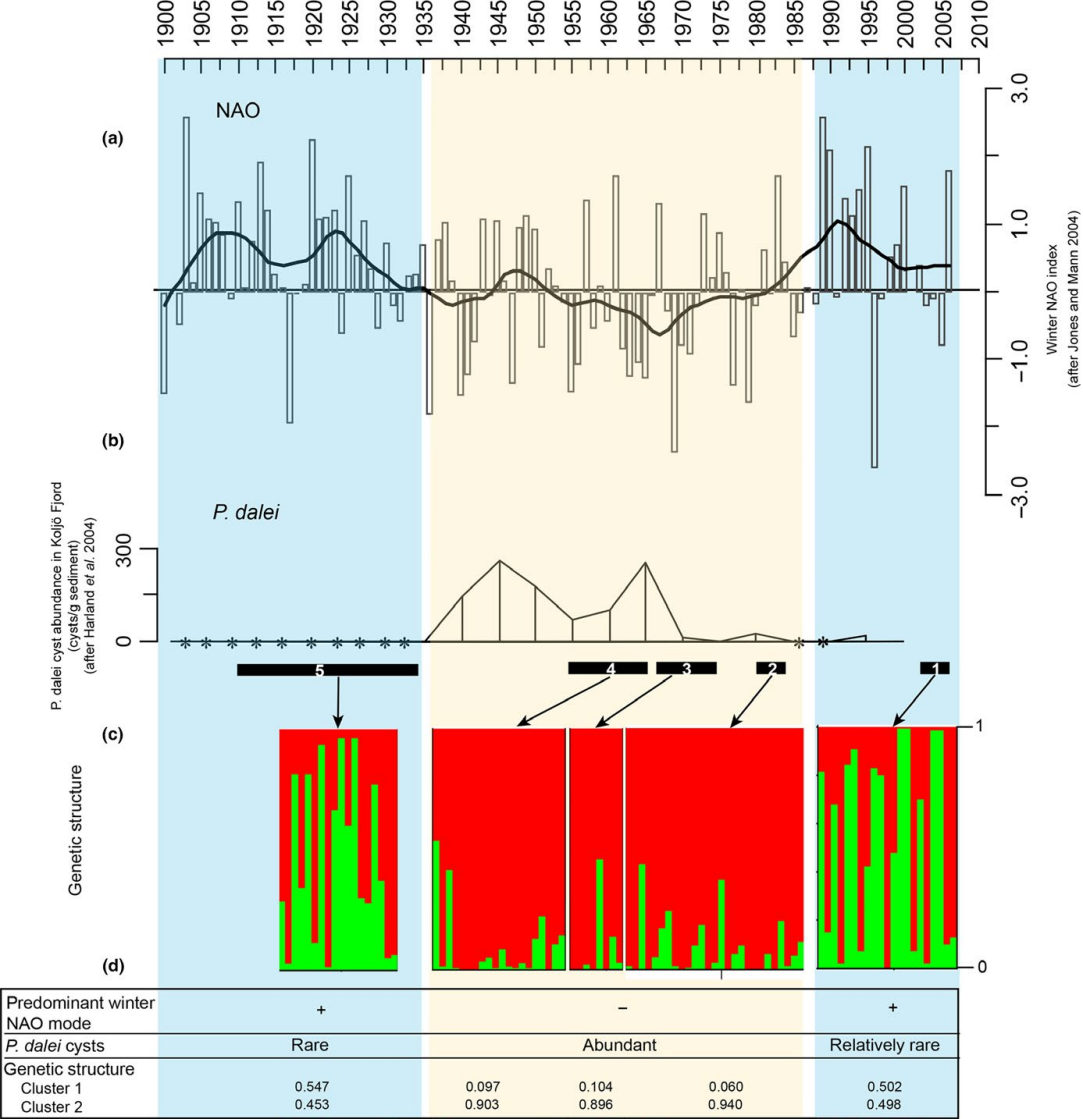
Comparison of population genetic structure of *Pentapharsodinium dalei* (c) with the winter NAO index variability since 1900 (a) and abundance of *P. dalei* cysts in Koljö Fjord (b) (details after Harland et al., 2004a, 2004b and Jones & Mann, 2004). *Denote cyst abundances below 1 cyst/g. Numbers 1–5 show the age range as estimated by ^210^Pb analyses for each of the sediment core layers from which resting cysts were isolated, germinated, and genotyped, that is, 2006; 1985 (±3 years); 1970 (±4 years); 1960 (±5 years); 1922 (±12 years). Bar plots of the population structure analysis were determined by Bayesian cluster analysis using STRUCTURE assuming no admixture, using sediment layer as prior, and with *K* = 2. Each vertical bar represents one individual and each color represents the fraction of the individuals assigned to each cluster. Table (d) summarizing predominant NAO mode, periods of high and low *P. dalei* abundances, and mean proportion of cluster membership. Clusters 1 and 2 indicate proportion of membership of each age depth in each of the two clusters

The STRUCTURE analyses including sample site information (LOCPRIOR) supported *K* = 2, when using the Δ*K* approach, and the proportion of the sample assigned to each cluster was asymmetric (≠1/*K* in each population, Figure 3). Among the three samples originating from 1960 to 1985, the individuals were strongly assigned to one of two clusters, indicating the existence of population structure. As illustrated in the bar plot (Figure 3), the major partition in the dataset was between the 1960 to 1985 subpopulation (Cluster 2), and a subpopulation comprising both the most recent, 2006, and the oldest layer, 1922 (Cluster1). The clustering was compared to abundances of *P. dalei* cysts in Koljö Fjord and indices of winter NAO over the same time period based on results from Harland et al. (2004a) and Jones and Mann (2004) (Figure 3).

The hierarchical AMOVA testing the potential substructure indicated by STRUCTURE (Cluster 1: subpopulations originating from 2006 and 1922; Cluster 2: subpopulations originating from 1960 to 1985) displayed that most of the variation was found within samples (99.03; Table 3). The amount of molecular variation found among clusters was significant at 2.13% while a negative value was found among samples within clusters, indicating no structure within the clusters. The corresponding *F*‐statistics were *F*_SC_ = −0.012 (among subpopulations within clusters) and *F*_CT_ = 0.021 (*p* < .05, among clusters). The AMOVA with no defined clusters partitioned 0.17% of the variation among sediment depths while the remaining 99.83% was partitioned among genotypes within depths (data not shown).

**Table 3:** Analysis of molecular variance within and among clusters identified by STRUCTURE (Cluster 1 subpopulations originating from 1922 and 2006; Cluster 2 subpopulation originating from 1960 to 1985) and within sediment layers (age depths)

Source of variation	Degrees of freedom	Sum of squares	Variance components	Percentage of variation
Among clusters	1	4.269	0.05268 Va	2.13^a^
Among age depths within clusters	3	5.845	−0.02862 Vb	−1.16
Within age depths	89	218.162	2.45126 Vc	99.03
Total	93	228.277	2.47531	

Fixation indices *F*_SC_ = −0.01182, *F*_ST_ = 0.00972, *F*_CT_ = 0.02128.

a *p* < .05.

## 4 Discussion

The sediment record that served as basis for this study has a robust age control, clear indication of undisturbed sediment deposition, and yielded a large number of well‐preserved viable resting stages of several protist species (Lundholm et al., 2011). Furthermore, multiproxy studies reconstructing the past fjord environment are available (Filipsson & Nordberg, 2004a; Filipsson et al., 2005; Harland et al., 2004a; Nordberg et al., 2001), making it a particularly well suited site for this type of study.

The STRUCTURE analyses indicated genetic differentiation occurring at a multidecadal scale, with the mid‐core strains (1960–1985) differing from the most recent (2006) and the oldest strains (1922). The mid‐core strains also had higher levels of genetic diversity especially in terms of allelic richness, and the number of private alleles suggesting that the mid‐core samples have more alleles at lower frequencies. The timing of these changes agrees with the well‐documented environmental changes in Koljö fjord, as the strains germinated from mid‐core layers correspond to a phase of predominantly negative winter NAO (1960, 1970) or variable winter NAO indices with low average values (1985), as opposed to the positive NAO phases corresponding to the bottom (ca. 1922) and topmost (2006) sediment core layers (Figure 3; Filipsson et al., 2005; Harland et al., 2004a; Nordberg et al., 2001). Studies in Koljö Fjord have shown that negative NAO indices correspond to relatively high bottom water salinities (>28.5) and the presence of a strong pycnocline. NAO is an expression of air pressure, and negative NAO in Scandinavia leads to cold and dry winters with mainly easterly–northeasterly winds and often presence of winter sea ice (Harland et al., 2004a; Nordberg et al., 2001). Further, high abundances of *P. dalei* cysts also in other fjords along the Swedish west coast correlate with negative NAO indices (Filipsson et al., 2005; Harland et al., 2004a).

The higher degree of genetic diversity in the middle section of the record and the changes in population substructure might therefore be explained by the environmental conditions driven by the NAO, resulting in one subpopulation performing better during negative NAO during which the bottom salinities are higher and the pycnocline stronger, and another doing better at positive NAO. During negative NAO, the high abundances of *P. dalei* have been suggested to be an indicator of the importance of the phytoplankton spring bloom (Harland et al., 2004a, 2004b). The indicated temporal differentiation of the fjord population thus appears to be environmentally and biologically meaningful. The pattern in STRUCTURE was supported by the AMOVA (*p* < .05), but not by pairwise *F*_ST_ estimates. We therefore suggest that *P. dalei* in Koljö Fjord exhibit an overall stability in population structure, which has persisted for almost a century. The stable population maintains an intrinsic substructure with two alternating subpopulations, which shift concomitantly with changes in the predominant environmental conditions.

### 4.1 Temporal variable natural selection

In aquatic protists, temporal genetic differentiation has mainly been studied over shorter time scales such as during a bloom, between blooms, or over days (Dia et al., 2014; Lebret et al., 2012; Richlen et al., 2012). The diatom Pseudo‐nitzschia multistriata was studied over a period of four consecutive years, and genetic differentiation was found between years, but a cyclic pattern in population genetic structure was also observed (Tesson et al., 2014), indicating that over time, populations or subpopulations at the same locality may vary depending of selection pressure due to environmental conditions.

Fluctuating selection pressure can drive the evolutionary mechanisms that generate phenotypic and genotypic variation (Chevin & Haller, 2014). For *P. dalei* in Koljö fjord, we found evidence of such mechanisms. The overall structure in neutral population genetic markers was stable over almost a century, but with recorded variations in population genetic diversity (see details above). Bell (2010) argued that agents of selection vary continually over time, that the direction of selection often changes, and that adaptive walks are often interrupted. This is hard to test, due to lack of long‐term data. However, the present study offers a system, which may serve the purpose. The large degree of genetic diversity and potential for both fluctuation and recovery over longer time scales documented here may help explain the long‐term success of aquatic protists. When environmental conditions change, rare genotypes may become common and/or population structuring may follow.

Aquatic protists forming “seed banks” have a genetic reservoir. Genetic differentiation may be explained by directional selection on certain phenotypes due to biotic or abiotic factors or delayed germination of certain cohorts of cysts. Short life cycles including a resting stage in combination with adaptation to the native habitat (Rengefors, Logares, Laybourn‐Parry, & Gast, 2015; Sjöqvist, Godhe, Jonsson, Sundqvist, & Kremp, 2015) suggests that over long periods, local subpopulations may alternate between periods of vegetative growth under optimal conditions in the water column and refugia in the sediment under suboptimal conditions. On a longer time scale, one may, however, consider that the temporally variable natural directional selection is balanced out, and our results suggest that overall population genetic diversity is maintained over large time scales. The sediment record based on neutral microsatellite markers from this study and the diatom *S. marinoi* from another Scandinavian fjord (Härnström et al., 2011) both indicate relative temporal stable genetic structure over a century for these marine protists.

### 4.2 Genetic diversity

Throughout the sediment record, a high diversity (*G*/*N* = 1, and average gene diversity of 0.83 ± 0.07 across loci) was found. This is in agreement with previous studies on populations of aquatic protists such as diatoms, dinoflagellates, coccolithophores, and raphidophytes in marine and freshwater environments, both in geographically restricted and in connected areas, with *G*/*N* ranging from 0.66 to 1.00 (Alpermann, Beszteri, John, Tillmann, & Cembella, 2009; Casteleyn et al., 2009, 2010; Erdner et al., 1996; Evans, Kühn, & Hayes, 2005; Godhe & Härnström, 2010; Härnström et al., 2011; Iglesias‐Rodriguez et al., 2006; Lebret et al., 2012; Lowe et al., 2010; Nagai et al., 2007, 2009; Richlen et al., 2012; Rynearson & Armbrust, 2000, 2004), confirming early studies based on a more limited number of phytoplankton strains (Hayhome, Whitten, Harkins, & Pfiester, 1987; Medlin et al., 1996; Skov, Lundholm, Pocklington, Rosendahl, & Moestrup, 1997).

This genetic diversity might be generated and maintained due to different factors. Sexual reproduction generates genetic recombination and is in cyst‐forming dinoflagellates typically linked to resting stage formation. We found no overall linkage disequilibrium in the dataset, indicating that sexual reproduction occurs regularly. Both sexual reproduction and resting stages may slow down the response to any directional selection, thereby maintaining genetic variation (Hairston, 1996).

Environmental heterogeneity (including biological interaction factors such as grazing and parasitism) also contributes to maintenance of genetic diversity (Bell, 2010; Gsell, Domis, Verhoeven, van Donk, & Ibelings, 2013). Indications that the effective population size of *P. dalei* are much smaller than total population size (Watts et al., 2013) raises questions of how high genetic diversity is maintained in *P. dalei*. Environmental heterogeneity in combination with the extensive vegetative propagation may counterbalance the otherwise anticipated effect of genetic drift on a small population. A previous study based on another set of strains spanning the same time period found a high degree of phenotypic variation (growth response to optimum salinities and pH tolerance) among the *P. dalei* strains both within age depths and maintained throughout a century (Ribeiro et al., 2013). This illustrates that the large genotypic variation found in this study correlates with a large phenotypic variation. Immigration is a third parameter, which may increase genetic diversity. However, Koljö Fjord is a sill fjord with limited water exchange and subsequent restrained horizontal migration, and the potential immigrants are probably outcompeted by locally adapted native populations (Godhe & Härnström, 2010; Härnström et al., 2011; Rynearson & Armbrust, 2000, 2004; Rynearson et al., 2006). Adaptation to local conditions may overrule gene flow and has been shown in competition experiments with *S. marinoi* isolated from Scandinavian offshore and fjord sites (Sildever, Sefbom, Lips, & Godhe, 2016).

As genetic diversity did not decrease linearly with age, we do not expect any bias due to culturing and impact on the comparison among age depths. As in all other population genetic studies on phytoplankton (e.g., Rynearson & Armbrust, 2000), a high proportion of the genotypes were unique (here 97.9%), illustrating the high level of genetic diversity.

### 4.3 Using resting stages for exploring responses to future environmental change

Emergence of resting stages may be thought of as migration from the past (Hairston & De Meester, 2008). Resting stages of organisms such as other dinoflagellates (Lundholm et al., 2011; Miyazono, Nagai, Kudo, & Tanizawa, 2012), diatoms (Härnström et al., 2011; McQuoid, Godhe, & Nordberg, 2002), haptophytes, and prasinophytes (Ellegaard et al., 2016) have been found to survive several decades in marine sediments. This study shows that viable resting stages of aquatic protists preserved in undisturbed sediments are valuable for determining temporal differentiation and exploring the impact of environmental changes on population genetic diversity over time. Another source of genetic data from the past is ancient DNA, which have been recovered in marine sediments back in time (Boere, Rijpstra, De Lange, Sinninghe Damsté, & Coolen, 2011; Klouch et al., 2016). During, for example, abrupt climate change scenarios, species possessing resting stages as a life‐cycle trait may survive periods of suboptimal conditions, whereas species without resting stages may not survive (e.g., Ribeiro et al., 2011). Resting stages thus make up a reservoir that can impact the response of the population, the species, and the community to environmental change. As shown in the present study, the availability of live resting stages of aquatic protists in undisturbed sediment provides a valuable source for exploring temporal differentiation and the impact of environmental changes on population diversity and stability. Using live resting stages may thus help to better understand and thus predict future responses of marine protists to different scenarios of climate and environmental disturbance.

It has been speculated that the mainly positive NAO phase seen since c. 1970 is related to increased greenhouse gas concentrations (Visbeck, Hurrel, Polvani, & Cullen, 2001). Such a scenario suggests that in the future, the *P. dalei* population will decrease in abundance and its genetic structure will resemble that of the 1920s and ca. 2006. However, since the beginning of 21st century, the winter NAO index has decreased in comparison with the 1990s (Figure 3), and there is still a large degree of uncertainty regarding future climate conditions in the North Atlantic, with an uncertain long‐term impact on primary producers and ecosystem functioning.

## Conflict of Interests

The authors declare no conflict of interests.

## Author Contribution

NL, AG, and ME contributed to research design. NL, SR, and ME performed research. NL and LRN analyzed the data. NL, SR, AG, LRN, and ME discussed results and contributed to written manuscript.

## Data Accessibility

Data available from the Dryad Digital Repository: doi:10.5061/dryad.195k3
