# Supplementary material for: Exploring the impact of multidecadal environmental changes on the population genetic structure of a marine primary producer
Source: Ecol Evol. 2017 Mar 30;7(9):3132–42. doi: 10.1002/ece3.2906 (PMC5415532; doi:10.1002/ece3.2906)

**Supplementary Figures**

# Fig. S1. Principal component analysis based on allele frequencies of six microsatellite loci of the five samples of cells germinated from 2006, 1985, 1970, 1960 and 1922 indicated by red, purple, green, blue and yellow color, respectively. The inserted eigenvalues illustrates the principal components of the data set.

#
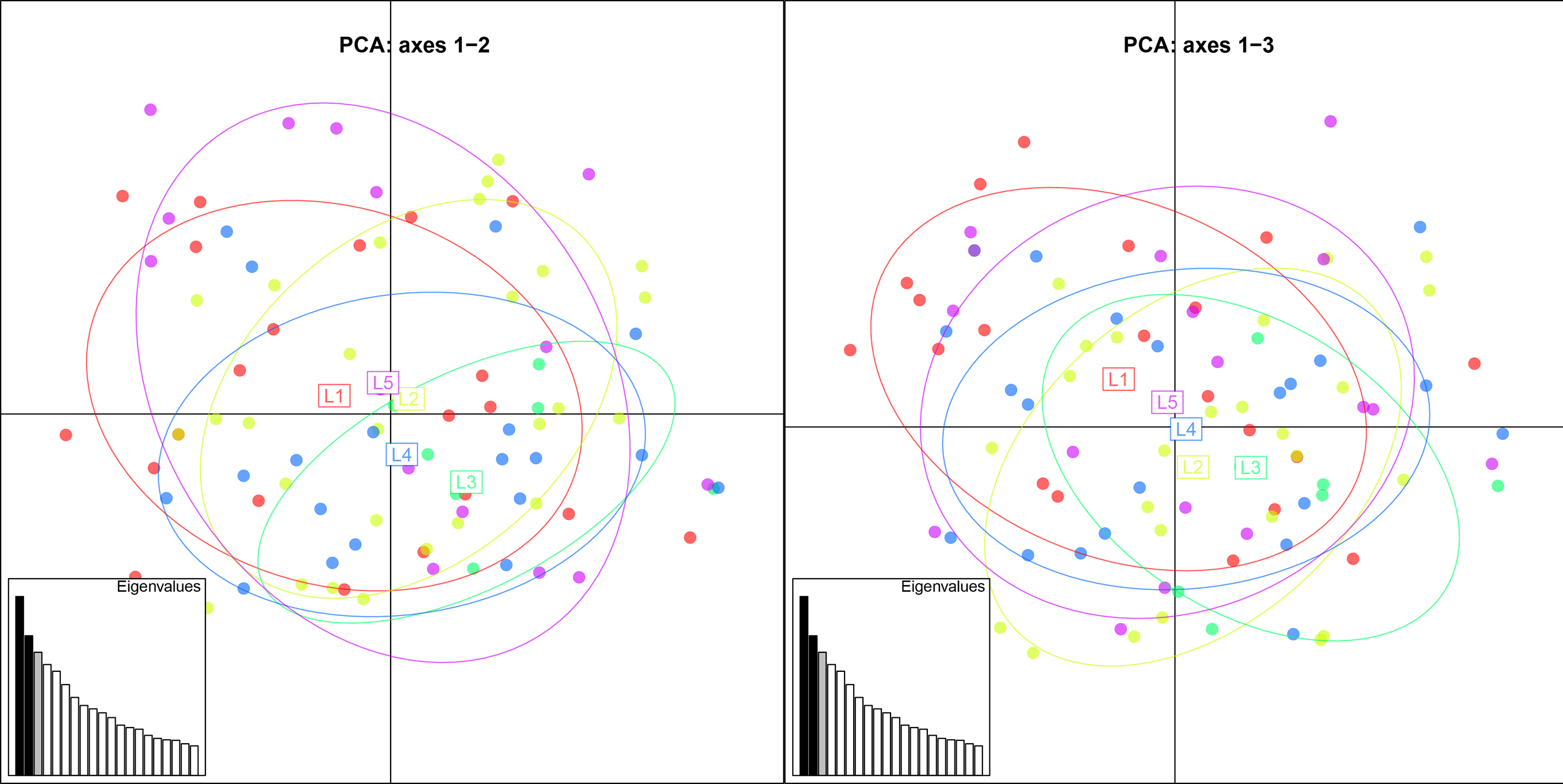

Supplement: Supplementary file 1 [file ECE3-7-3132-s001.docx]
